# Is It Necessary To Add Soft Tissue Injury to the Classification in Tibial Plateau Fracture Management?

**DOI:** 10.7759/cureus.22236

**Published:** 2022-02-15

**Authors:** Mahmut Tuncez, Ihsan Akan, Fırat Seyfettinoğlu, Hülya Çetin Tunçez, Berna Dirim Mete, Cemal Kazımoğlu

**Affiliations:** 1 Department of Orthopedics and Traumatology, Izmir Katip Celebi University Ataturk Training and Research Hospital, Izmir, TUR; 2 Department of Orthopedics and Traumatology, Adana City Training and Research Hospital, Adana, TUR; 3 Department of Radiology, Bilecik Training and Research Hospital, Bilecik, TUR; 4 Department of Radiology, Izmir Democracy University Faculty of Medicine, Izmir, TUR

**Keywords:** anterior cruciate ligament, meniscus, magnetic resonance imaging, classification, tibial plateau fracture

## Abstract

Background

A gold standard classification for the treatment of tibial plateau fractures with soft tissue injury has not been established yet.This study aimed to evaluate the usability of a novel modified classification that can provide preoperative information to the surgeon about soft tissue injuries in tibial plateau fractures.

Methodology

A total of 36 patients with tibial plateau fractures were included in the study. Patients’ age, gender, and affected sides were recorded. Injuries to the medial meniscus, lateral meniscus, anterior cruciate ligament, posterior cruciate ligament, medial collateral ligament, and lateral collateral ligament were examined with preoperative magnetic resonance imaging. Soft tissue injuries were arranged according to the novel modified classification based on the Schatzker classification.

Results

The mean age of the study participants was 45 (19-76) years; 72% of the patients were men and 28% were women. Moreover, 44% and 56% of the patients had broken the right and left tibial plateaus, respectively. At least one soft tissue injury was detected in 29 (81%) patients. In 14 (39%) patients, two or more soft tissue injuries were observed. All patients were arranged according to the novel modified classification regarding ligament and meniscus injuries.

Conclusions

With this novel modified classification system, we think that having better information about the preoperative condition of the soft tissue injuries can change the surgical strategy in patients with tibial plateau fractures.

## Introduction

Tibial plateau fractures are intra-articular complex injuries with a wide clinical and radiological spectrum. Postoperative soft tissue complications and knee dysfunction are likely to develop in these fractures [1]. Following a high-energy shear and compressive force on the knee joint, 39-99% of the patients are at risk of injury to the meniscus, collateral ligaments, and cruciate ligaments [2-5]. In the emergency department, these patients are traditionally diagnosed with standard anteroposterior and lateral knee radiography and computed tomography (CT). CT provides more optimum information of the bone tissues than other imaging modalities to predict and reconstruct the fracture pattern. However, CT does not provide enough preoperative information about soft tissue injuries around the knee after trauma. In recent years, magnetic resonance imaging (MRI) has been used in tibial plateau fractures because it provides better quality, allows detailed examination of the soft tissues, shows the amount of fragmentation, and can show hidden fracture lines [4,6,7].

Current classification systems have generally been developed based on the type of fracture patterns. The most commonly used is the Schatzker classification system, which includes six types of tibial plateau fractures [8]. While this classification evaluates the fracture in two dimensions, three-dimensional classifications were introduced after the spread of CT. To our knowledge, the classification system regarding soft tissue injuries based on MRI findings has not been established yet. Thus, this study aimed to evaluate the usability of a novel modified classification that can provide preoperative information to the surgeon about soft tissue injuries in tibial plateau fractures.

## Materials and methods

This retrospective study was approved by the Institutional Review Board of our hospital. Between January 2018 and December 2019, 36 of 43 patients who were admitted to our hospital with a diagnosis of tibial plateau fracture and received surgical treatments were included in the study.

Seven patients who could not undergo MRI for various reasons (cardiac stent, claustrophobia, etc.) were not included in the study. Anteroposterior and lateral knee X-ray imaging and knee CT were routinely performed when patients were admitted to the emergency department. Preoperative MRI was also routinely obtained for all patients, and soft tissue injuries were noted. The following patients were excluded from the study: those who underwent emergency surgery because of open fractures and circulatory disorders, those who could not undergo MRI because of claustrophobia, those who were treated conservatively (tibial plateau fractures with nondisplaced displacement of less than 2 mm and avulsion injury) and discharged from the emergency department, those with an immature skeletal system, and those aged <18 years. Data concerning demographics, injury mechanism, age, gender, and injured side were noted in all patients. All tibial plateau fractures were evaluated preoperatively by a single radiologist (BDM) and a single orthopedist (MT) according to the Schatzker classification. Statistically, demographic data and MRI findings were evaluated by correlating them. In addition, the distribution of soft tissue injuries according to fracture types was examined.

Knee MRI examination was performed using a 1.5-Tesla MRI device (General Electric Medical Systems, Milwaukee, WI, USA) using a knee coil. For all cases, MRI was applied using standard sagittal proton density fat-suppressed turbo spin-echo (TSE) sequences (repetition time/echo time [TR/TE], 1,500/15; matrix, 256 × 384; field of view [FOV], 18 cm), coronal proton density fat-suppressed TSE (TR/TE, 1,500/15; matrix, 256 × 320; FOV, 18 cm), coronal T1-weighted TSE (TR/TE, 500/18; matrix, 256 × 320; FOV, 21 cm), and axial T2-weighted fat-suppressed TSE (TR/TE, 2000/60; matrix, 256 × 288; FOV, 16 cm). The slice thickness was 3 mm. The knee MR images were evaluated by a musculoskeletal radiologist with 20 years of experience. For statistical analysis, the percentage of each soft tissue injury was calculated based on the total number of fractures and determined in percentages according to the Schatzker classification.

Modification of classification

**Figure 1:**
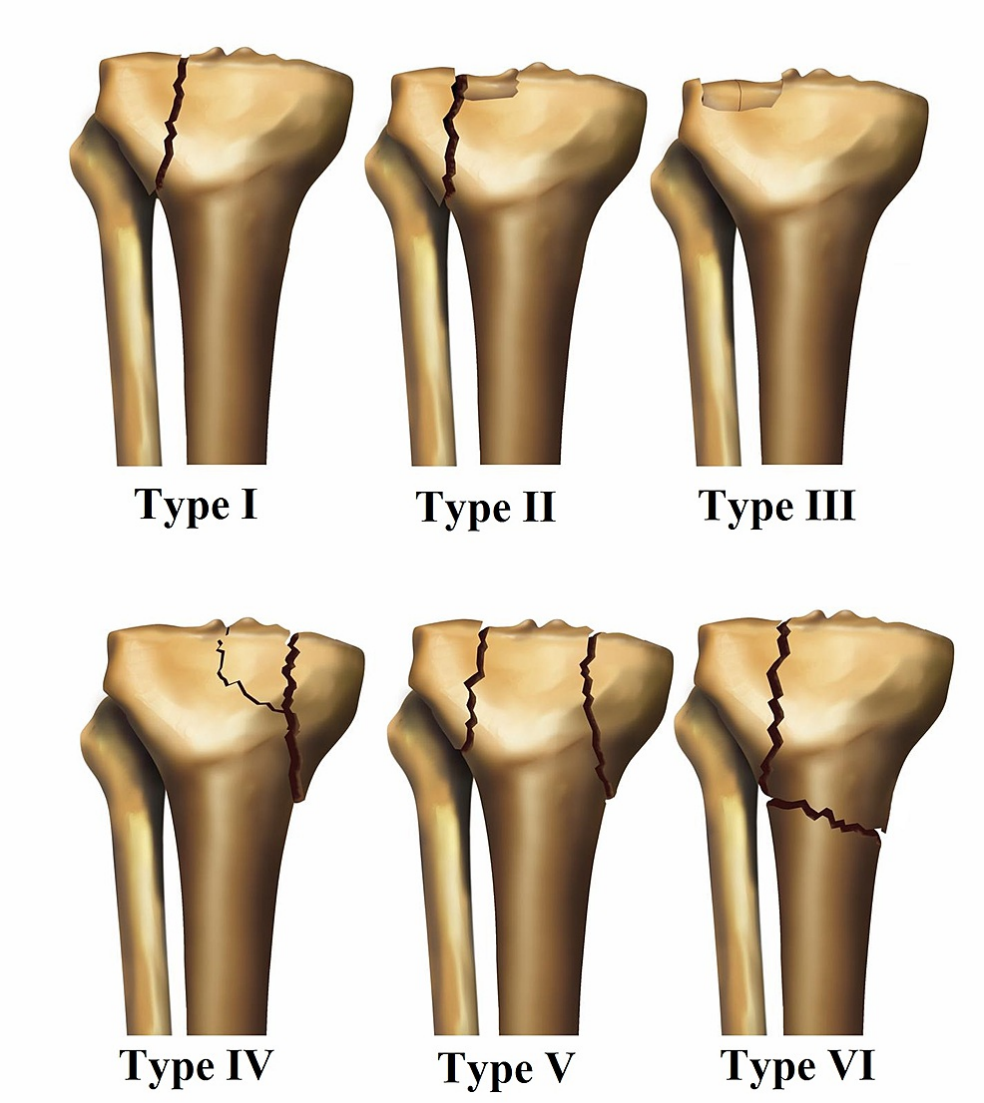
Schatzker classification.

Schatzker had originally described his classification in 1974, presenting six types of plateau fractures [8]. Type I is a pure cleavage fracture of the lateral tibial plateau (Figure 1). Type II is a combined cleavage and compression fracture of the lateral plateau. Type III is pure compression of the lateral plateau. Types IV-VI are high-energy injuries associated with knee joint instability from subluxation to dislocation. Type IV is an isolated fracture of the medial column of the tibial plateau. Type V fracture is a bicondylar fracture in which the continuity of the shaft is preserved with the overlying metaphysis and part of the joint. In type VI, joint fractures also involve the tibial metaphysis.

**Figure 2:**
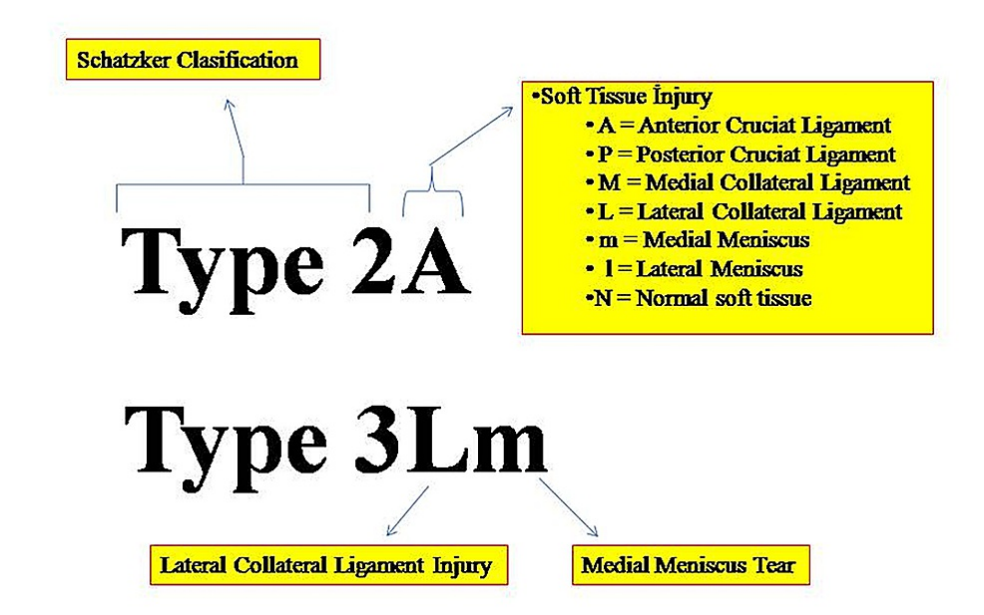
The novel modified classification based on the Schatzker classification.

**Figure 3:**
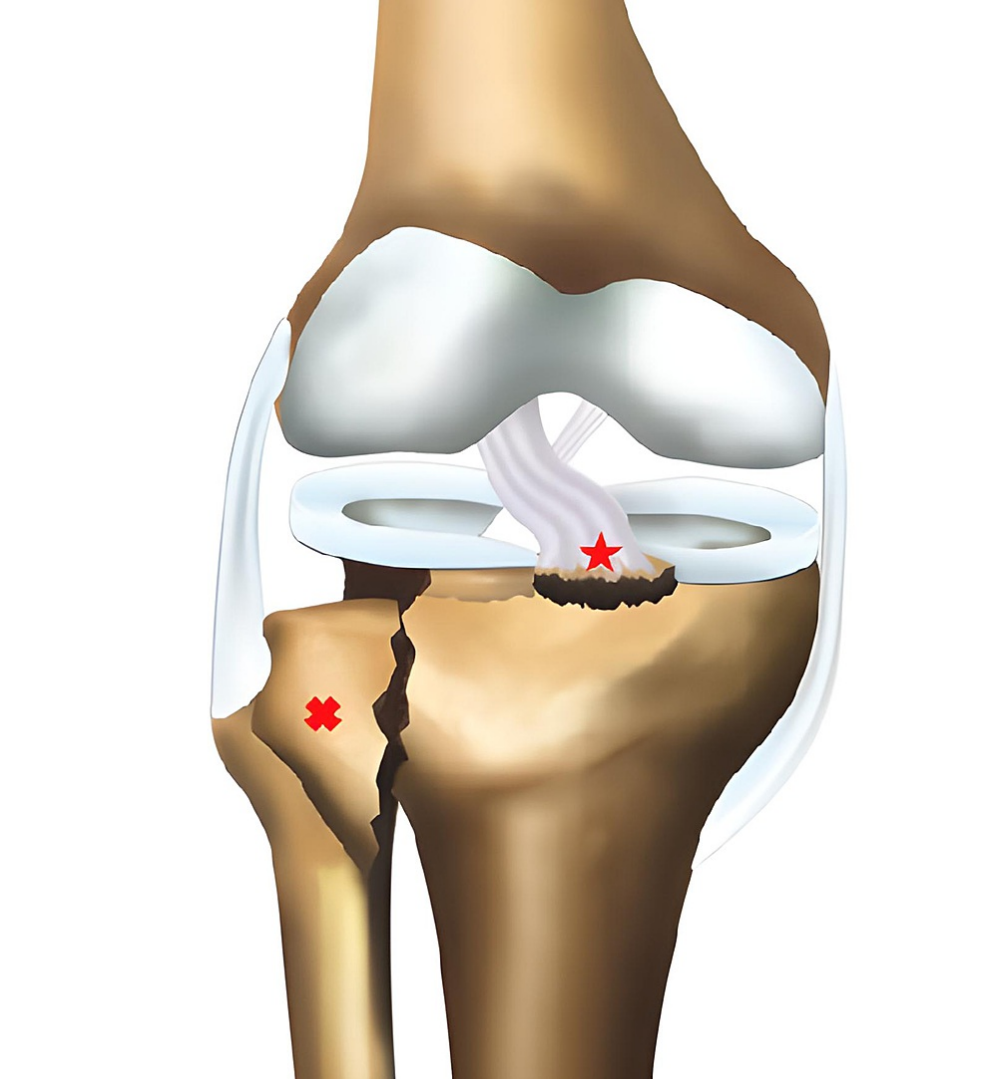
Type 2A fracture according to the new classification; the cross sign shows Schatzker type 2 fracture, and the star sign shows anterior cruciate ligament avulsion fracture.

**Figure 4:**
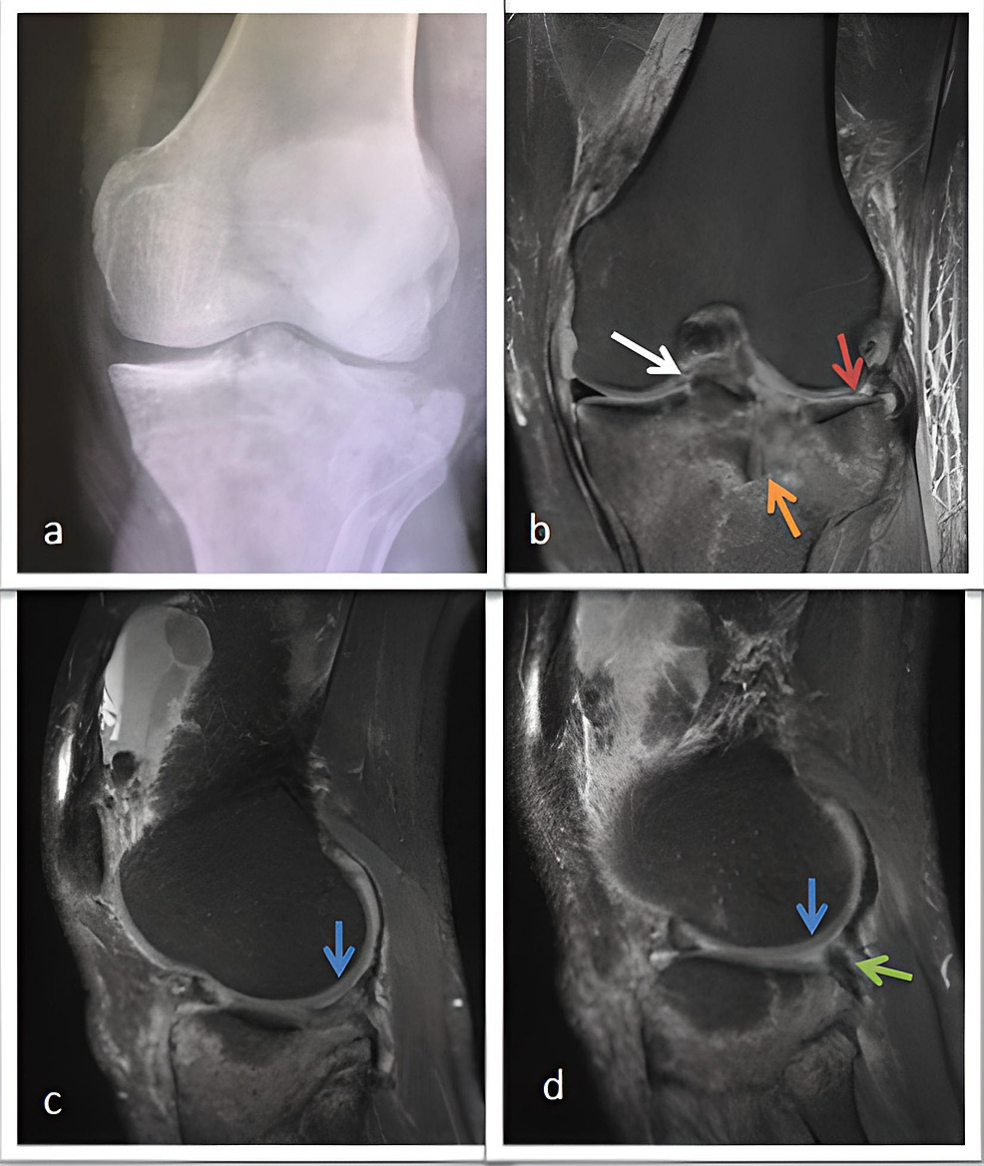
A 43-year-old male patient with a type 2Al fracture according to the novel modified classification. (a) X-ray of type 2 tibial plateau fracture according to the Schatzker classification. (b) FS-PD coronal MRI shows the absence of the corpus of the lateral meniscus (red arrow) due to bucket-handle tear. The white arrow shows the footprint bone avulsion of the ACL, and the orange arrow shows the displaced osteochondral joint surface (c-d) FS-PD sagittal MRI shows the bucket-handle tear of lateral meniscus, the blue arrow shows the absence of the posterior horn of the lateral meniscus, and the green arrow shows the popliteus tendon. FS/PD: fat-suppressed/proton density; ACL: anterior cruciate ligament; MRI: magnetic resonance imaging

**Figure 5:**
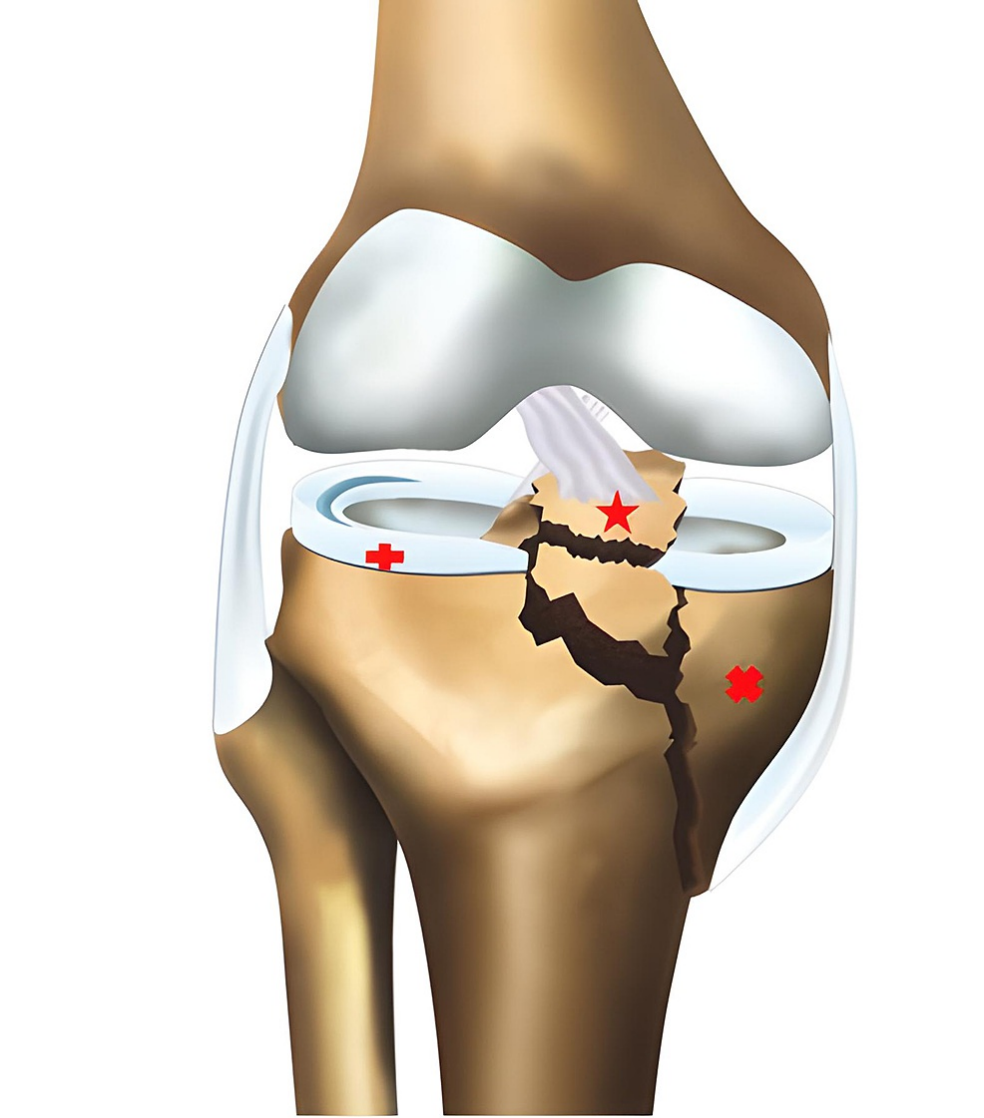
Type 4Al fracture according to the novel modified classification; the cross sign shows Schatzker type 4 fracture, the star sign shows anterior cruciate ligament avulsion fracture, and the plus sign shows grade 3 lateral meniscal tear.

According to the current literature, the risk of accompanying soft tissue injuries is comparatively higher in tibial plateau fractures. In our view, obtaining detailed information about concomitant soft tissue injuries plays a critical role in the management of these complex injuries. Therefore, we modified the Schatzker classification by adding letters that show the preoperative soft tissue injuries in these fractures (Figure 2). We preferred adding “A” for patients who had anterior cruciate ligament (ACL) injury (Figure 3). Treatment can be planned accordingly. We can use “P” for posterior cruciate ligament (PCL) injury, “L” for lateral collateral ligament (LCL) injury, “M” for medial collateral ligament (MCL) injury, “m” for medial meniscus injury, and “l” for lateral meniscus injury (Figure 4). The letters can be used together for multiple tissues. For example, type IVAl (ACL and lateral meniscus injury with a Schatzker type IV fracture) (Figure 5). “N” can be preferred for those who do have soft tissue injuries. Overall, this presents the modified Schatzker classification system based on preoperative MRI findings.

## Results

This study involved 36 patients, including 26 (72%) men and 10 (28%) women. The average age of the patients was 45 (19-76) years. Moreover, 16 (44%) patients had right tibial plateau fractures, and 20 (56%) had left tibial plateau fractures. The causes of fractures due to high-energy injuries included motor vehicle accidents, falling from a height, and sports injuries. According to the Schatzker classification, six type I (pure split), eleven type II (split combined with depression), six type III (pure depression), five type IV (medial plateau), three type V (bicondy), and five type VI (plateau and metaphysis) fractures were present (Table 1). At least one soft tissue injury was detected in 29 (81%) patients. In 14 (39%) patients, two or more soft tissue injuries were observed (Table 2).

**Table 1:** Schatzker classification and distribution of soft tissue injury. MM: medial meniscus; LM: lateral meniscus; MCL: medial collateral ligament; LCL: lateral collateral ligament; ACL: anterior cruciate ligament; PCL: posterior cruciate ligament

Schatzker classification	Number of patients	LM injury	MM injury	ACL injury	PCL injury	LCL injury	MCL injury	Bucket-handle meniscus injury
I	6	2	3	2	0	0	0	1
II	11	4	1	7	2	0	0	2
III	6	2	0	2	0	0	0	0
IV	5	0	1	2	2	0	0	0
V	3	1	1	1	1	0	0	0
VI	5	2	1	3	1	0	0	1

**Table 2:** Patient data and tibial plateau fracture classification distributions. M: male; F: female; R: right; L: left; MM: medial meniscus; LM: lateral meniscus; MCL: medial collateral ligament; LCL: lateral collateral ligament; ACL: anterior cruciate ligament; PCL: posterior cruciate ligament

Patient	Sex	Age	Side	AO classification	Schatzger classification	MM injury	LM injury	MCL injury	LCL injury	ACL injury	PCL injury	Bucket-handle meniscus tear	New modified classification
1	M	27	R	B3	2	-	-	-	-	-	-	-	TİP 2N
2	F	68	R	B3	1	-	+	-	-	+	-	+	TİP 1Al
3	F	22	R	C2	4	-	-	-	-	-	-	-	TİP 4N
4	M	43	L	B3	2	-	+	-	-	+	-	+	TİP 2Al
5	F	76	L	B2	3	-	+	-	-	-	-	-	TİP 3l
6	M	33	L	B1	1	-	+	-	-	-	-	-	TİP 1l
7	M	30	R	B2	2	-	+	-	-	-	-	-	TİP 2l
8	F	57	L	B3	2	-	+	-	-	+	-	+	TİP 2Al
9	M	70	L	C1	4	+	-	-	-	-	-	-	TİP 4m
10	M	31	L	B1	1	+	-	-	-	-	-	-	TİP 1m
11	M	32	L	B1	1	+	-	-	-	+	-	-	TİP 1Am
12	F	54	L	B1	3	-	-	-	-	+	-	-	TİP 3A
13	M	42	L	B3	2	-	-	-	-	+	+	-	TİP 2AP
14	M	33	L	B3	3	-	-	-	-	-	-	-	TİP 3N
15	M	42	R	C3	5	-	-	-	-	-	-	-	TİP 5N
16	M	19	R	C3	6	-	-	-	-	-	+	-	TİP 6P
17	M	63	L	B2	3	-	-	-	-	-	-	-	TİP 3N
18	F	67	L	C1	4	-	-	-	-	-	+	-	TİP 4P
19	M	30	R	C3	6	-	+	-	-	+	-	-	TİP 6Al
20	M	51	R	C1	4	-	-	-	-	+	+	-	TİP 4AP
21	F	56	L	B2	3	-	+	-	-	-	-	-	TİP 3Al
22	F	68	R	B3	2	+	-	-	-	-	-	-	TİP 2m
23	M	55	L	B3	2	-	-	-	-	+	-	-	TİP 2A
24	M	30	R	B2	3	-	-	-	-	+	-	-	TİP 3A
25	M	27	L	B1	1	+	-	-	-	-	-	-	TİP 1m
26	M	57	L	C3	6	+	+	-	-	-	-	+	TİP 6lm
27	M	38	R	B1	1	-	-	-	-	-	-	-	TİP 1N
28	M	29	R	B3	2	-	-	-	-	+	+	-	TİP 2AP
29	M	33	L	B3	2	-	+	-	-	+	-	-	TİP 2Al
30	M	48	R	B3	2	-	-	-	-	-	-	-	TİP 2N
31	M	47	L	B3	2	-	-	-	-	+	-	-	TİP 2A
32	F	53	L	C3	6	-	+	-	-	+	-	-	TİP 6Al
33	M	32	R	C1	4	-	-	-	-	+	-	-	TİP 4A
34	F	74	L	C2	5	+	-	-	-	-	-	-	TİP 5m
35	M	52	R	C3	6	-	-	-	-	+	-	-	TİP 6Al
36	M	36	R	C3	5	-	+	-	-	+	+	-	TİP 5APl

Anterior cruciate ligament/Posterior cruciate ligament

Cruciate ligament injuries were classified as partial or total injuries. The reduction of the ligament thickness and high signal intensity within the ligament was accepted as partial tears. Total cruciate ligament injuries were also divided into two groups, namely, bony avulsion injuries and complete disruption of ligament integrity. In addition, five (14%) patients had no cruciate ligament injuries, six (17%) had partial tears, and 25 (69%) had bone avulsion type ACL injury. ACL bone avulsion injuries were evaluated with CT and MRI and grouped according to the Meyers and McKeever classification. In this classification, type I refers to nondisplaced fractures, type II to anterior displaced fractures, type III to fully displaced fractures, and type IV (later added by Zariczynj) to comminuted fractures [9,10]. Accordingly, eight type I, ten type II, two type III, and five type IV bone avulsions were found. Types II-IV were included in the novel modified classification that included soft tissue injuries as they may require surgical intervention [11,12]. Partial tears and type I bone avulsions are not included in the soft tissue classification as they can be followed conservatively. PCL bone avulsion was observed in six (17%) patients.

Medial meniscus/Lateral meniscus

The Stoller classification system was used in the evaluation of the meniscus on MRI [13]. On MRI, cases with grades 1 and 2 were accepted as the group with no meniscus tear, and those with grade 3 as the group with a meniscus tear. The point signal in the meniscus was evaluated as grade 1, linear signal as grade 2, and signal extending to the joint surface with displaced tears, such as bucket-handle tears, as grade 3.

When the medial meniscuses were examined, 11 (31%) patients had grade 1, 18 (50%) had grade 2, and seven (19%) had grade 3 injuries. In the lateral meniscus, six (17%) patients had grade 1, 17 (47%) had grade 2, and 13 (36%) had grade 3 injuries. As the new classification in our study may require surgery, grade 3 lesions were taken into account (12). Four of these meniscus injuries were bucket-handle tears.

Medial collateral ligament/Lateral collateral ligament

MCL and LCL injuries were classified as partial and complete. We did not encounter totally ruptured MCL and LCL in our patients. However, 31 (86%) patients had partial MCL injuries. LCL injury was also detected in 31 (86%) patients. In our novel modified classification, totally ruptured lesions were taken into consideration because of the need for surgical reconstruction.

## Discussion

Despite the known value of MRI in determining pathologies of the ligaments and meniscus, some studies have shown that bone fragments are also well defined in tibial plateau fractures in MRI [2,6,14,15]. Optimum preoperative planning can be improved when the surgeon is aware of concomitant soft tissue injuries [6,16-18]. Currently, despite attempts to evaluate soft tissue injuries with arthroscopy-guided procedures, it cannot be performed acutely in every type of fracture, and sufficient information about the PCL and posterior soft tissue components cannot be obtained [19,20]. In routine practice, although the importance of soft tissue injuries in the treatment of tibial plateau fractures is known, its treatment is focused on the fractures. A surgeon who is fully aware of the ligament and meniscus injury preoperatively cannot ignore this injury. Although soft tissue injury is accompanied by up to 99% of tibial plateau fractures, we found at least one soft tissue injury in 81% (n = 29) of our patients. In 39% (n = 14), two or more ligament and meniscus lesions were observed. Additionally, we detected PCL injuries (n = 6) that cannot be detected by routine arthroscopy.

Audigé et al. emphasized that a classification system should be understandable, documentable, and usable between observers. The types described should apply to real cases that often occur in practice [21]. Although many classification systems have been published for tibial plateau fractures, the Schatzker classification and Arbeitsgemeinschaft für Osteosynthesefragen (AO)/Orthopedic Trauma Association (OTA) are the most studied ones because of their reliability. When the Schatzker classification increases numerically, it reflects not only the increase in energy delivered to the bone at the time of injury but also a poor prognosis [22]. Therefore, orthopedic surgeons find the Schatzker classification useful in assessing initial injury, planning, and predicting prognosis [22]. In most reliability studies using only plain radiographs, the reliability of the Schatzker and AO/OTA classification systems was rated poor or moderate [23,24]. Several authors have reported that the initial Schatzker classification and surgical plans were changed based on plain radiographic findings after preoperative three-dimensional imaging (CT or MRI) [6,25,26].

In their study, Yacoubian et al. used CT and MRI separately in the classification of tibial plateau fractures. They showed that the treatment plan was changed in 23% of the cases where MRI was used preoperatively, and the classification was also changed in 21% of the cases [6]. Preoperatively, they suggested that MRI can be superior to CT. In addition, meniscus injury, ligament injury, cartilage displacement, and the number of cartilage fragments can be determined with MRI. With the proposed new classification, the orthopedic surgeon is aware of these injuries beforehand and can follow the soft tissues during the planned operation for fracture fixation or during secondary surgery. Although CT evaluation is important, especially in preoperative planning of plate placement and the direction of the screws, we think that MRI is also important in changing the treatment strategy. In our study, displaced bucket-handle tears were detected in the medial meniscus in one patient and the lateral meniscus in three patients. Arthroscopic meniscus repair was performed in these patients.

Delamarter et al. concluded that the delayed reconstruction of collateral ligaments and cruciate ligaments is crucial to maintain knee stability and reduce overall morbidity [27]. Moreover, many authors have emphasized the importance of acute fixation of ACL bony avulsions [28,29]. In our study, we detected ACL injury that may require surgery in 47% of the patients. In addition, in 17% of the patients, we found PCL bone avulsion displaced >2 mm. We believe that the use of MRI in the preoperative detection of soft tissue injuries in the posterior part of the knee may contribute significantly to the clinical outcome.

In their study, Cinque et al. did not find a significant difference in the clinical presentation of patients who underwent surgery for isolated ligament injury and tibial plateau fractures [30]. However, some researchers argue that acute repair does not give good clinical results in tibial plateau fractures and recommend reconstruction during the second operation [27]. In both cases, knowledge of soft tissue injuries will benefit the patient and the surgeon in clinical outcomes.

Limitations

Patients with minimal displacement and those requiring conservative treatment were excluded, the study prospectively lacked clinical results based on MRI classification, and findings were not routinely supported by perioperative arthroscopy.

## Conclusions

We believe that the proposed modified classification system will provide better information about the condition of soft tissue injuries preoperatively and thereby can change the surgical strategy. As a result, adding soft tissue injuries based on preoperative MRI findings to the classification will contribute to clinical and functional results in tibial plateau fractures. We believe that this modification, based on the Schatzker classification, will provide a guideline for a better understanding of the complex nature of these injuries and an accurate preoperative treatment plan. In addition, it will improve the exchange of information among observers and future studies on bone and soft tissue injuries.

## References

[1] Kfuri M, Schatzker J (2018). Revisiting the Schatzker classification of tibial plateau fractures. Injury.

[2] Kode L, Lieberman JM, Motta AO, Wilber JH, Vasen A, Yagan R (1994). Evaluation of tibial plateau fractures: efficacy of MR imaging compared with CT. AJR Am J Roentgenol.

[3] Mustonen AO, Koivikko MP, Lindahl J, Koskinen SK (2008). MRI of acute meniscal injury associated with tibial plateau fractures: prevalence, type, and location. AJR Am J Roentgenol.

[4] Gardner MJ, Yacoubian S, Geller D (2005). The incidence of soft tissue injury in operative tibial plateau fractures: a magnetic resonance imaging analysis of 103 patients. J Orthop Trauma.

[5] Chang H, Zheng Z, Shao D, Yu Y, Hou Z, Zhang Y (2018). Incidence and radiological predictors of concomitant meniscal and cruciate ligament injuries in operative tibial plateau fractures: a prospective diagnostic study. Sci Rep.

[6] Yacoubian SV, Nevins RT, Sallis JG, Potter HG, Lorich DG (2002). Impact of MRI on treatment plan and fracture classification of tibial plateau fractures. J Orthop Trauma.

[7] Evangelopoulos D, Chalikias S, Michalos M (2020). Medium-term results after surgical treatment of high-energy tibial plateau fractures. J Knee Surg.

[8] Schatzker J (1974). Compression in the surgical treatment of fractures of the tibia. Clin Orthop Relat Res.

[9] Meyers MH, McKeever FM (1959). Fracture of the intercondylar eminence of the tibia. J Bone Joint Surg Am.

[10] Zaricznyj B (1977). Avulsion fracture of the tibial eminence: treatment by open reduction and pinning. J Bone Joint Surg Am.

[11] Seon JK, Park SJ, Lee KB (2009). A clinical comparison of screw and suture fixation of anterior cruciate ligament tibial avulsion fractures. Am J Sports Med.

[12] Biyani A, Reddy NS, Chaudhury J, Simison AJ, Klenerman L (1995). The results of surgical management of displaced tibial plateau fractures in the elderly. Injury.

[13] Stoller DW, Martin C, Crues JV 3rd, Kaplan L, Mink JH (1987). Meniscal tears: pathologic correlation with MR imaging. Radiology.

[14] Barrow BA, Fajman WA, Parker LM, Albert MJ, Drvaric DM, Hudson TM (1994). Tibial plateau fractures: evaluation with MR imaging. Radiographics.

[15] Ruth JT (2001). Fractures of the tibial plateau. Am J Knee Surg.

[16] Shepherd L, Abdollahi K, Lee J, Vangsness CT Jr (2002). The prevalence of soft tissue injuries in nonoperative tibial plateau fractures as determined by magnetic resonance imaging. J Orthop Trauma.

[17] Rossi R, Bonasia DE, Blonna D, Assom M, Castoldi F (2008). Prospective follow-up of a simple arthroscopic-assisted technique for lateral tibial plateau fractures: results at 5 years. Knee.

[18] Christiano AV, Pean CA, Kugelman DN, Konda SR, Egol KA (2020). Function and knee range of motion plateau six months following lateral tibial plateau fractures. J Knee Surg.

[19] Fowble CD, Zimmer JW, Schepsis AA (1993). The role of arthroscopy in the assessment and treatment of tibial plateau fractures. Arthroscopy.

[20] Vangsness CT Jr, Ghaderi B, Hohl M, Moore TM (1994). Arthroscopy of meniscal injuries with tibial plateau fractures. J Bone Joint Surg Br.

[21] Audigé L, Bhandari M, Hanson B, Kellam J (2005). A concept for the validation of fracture classifications. J Orthop Trauma.

[22] Markhardt BK, Gross JM, Monu JU (2009). Schatzker classification of tibial plateau fractures: use of CT and MR imaging improves assessment. Radiographics.

[23] Maripuri SN, Rao P, Manoj-Thomas A, Mohanty K (2008). The classification systems for tibial plateau fractures: how reliable are they?. Injury.

[24] Charalambous CP, Tryfonidis M, Alvi F, Moran M, Fang C, Samarji R, Hirst P (2007). Inter- and intra-observer variation of the Schatzker and AO/OTA classifications of tibial plateau fractures and a proposal of a new classification system. Ann R Coll Surg Engl.

[25] Wicky S, Blaser PF, Blanc CH, Leyvraz PF, Schnyder P, Meuli RA (2000). Comparison between standard radiography and spiral CT with 3D reconstruction in the evaluation, classification and management of tibial plateau fractures. Eur Radiol.

[26] Macarini L, Murrone M, Marini S, Calbi R, Solarino M, Moretti B (2004). Tibial plateau fractures: evaluation with multidetector-CT. Radiol Med.

[27] Delamarter RB, Hohl M, Hopp E Jr (1990). Ligament injuries associated with tibial plateau fractures. Clin Orthop Relat Res.

[28] Wang Z, Tang Z, Liu C, Liu J, Xu Y (2017). Comparison of outcome of ARIF and ORIF in the treatment of tibial plateau fractures. Knee Surg Sports Traumatol Arthrosc.

[29] Kampa J, Dunlay R, Sikka R, Swiontkowski M (2016). Arthroscopic-assisted fixation of tibial plateau fractures: patient-reported postoperative activity levels. Orthopedics.

[30] Cinque ME, Godin JA, Moatshe G, Chahla J, Kruckeberg BM, Pogorzelski J, LaPrade RF (2017). Do tibial plateau fractures worsen outcomes of knee ligament injuries? A matched cohort analysis. Orthop J Sports Med.

